# Physical Measures to Reduce Exposure to Tap Water–Associated Nontuberculous Mycobacteria

**DOI:** 10.3389/fpubh.2020.00190

**Published:** 2020-06-12

**Authors:** Grant J. Norton, Myra Williams, Joseph O. Falkinham, Jennifer R. Honda

**Affiliations:** ^1^Department of Biomedical Research, Center for Genes, Environment and Health, National Jewish Health, Denver, CO, United States; ^2^Department of Biological Sciences, Virginia Tech, Blacksburg, VA, United States

**Keywords:** *Mycobacterium smegmatis*, *Mycobacterium abscessus*, *Mycobacterium avium*, *Mycobacterium chimaera*, tap water, point of use filters, UV disinfection, biofilm

## Abstract

Nontuberculous mycobacteria (NTM) that cause human disease can be isolated from household tap water. Easy-to-use physical methods to reduce NTM from this potential source of exposure are needed. Filters and UV disinfection have been evaluated for their ability to reduce numbers of waterborne non-NTM organisms from drinking water, but their efficacy in reducing NTM counts are not well-established. Thus, five commercially available disinfection methods were evaluated for their potential as practical, efficient, and low-cost methods to reduce NTM from tap water. First, suspensions of tap water–adapted *Mycobacterium smegmatis* were passed through either a point-of-use, disposable, 7-day or 14-day Pall-Aquasafe filter. The 7-day filter prevented passage of *M. smegmatis* in effluent water for 13 days, and the 14-day filter prevented the passage of *M. smegmatis* for 25 days. Second, a granular activated carbon filter system failed to significantly reduce *Mycobacterium abscessus* and *Mycobacterium avium* numbers. Third, suspensions of tap water–adapted *M. abscessus, M. avium*, and *M. chimaera* (“MycoCocktail”) were passed through the “LifeStraw GO” hollow-fiber, two-stage membrane filtration system. LifeStraw GO prevented passage of the MycoCocktail suspension for the entire 68-day evaluation period. Finally, two different water bottle UV sterilization systems, “Mountop” and “SteriPEN,” were evaluated for their capacity to reduce NTM numbers from tap water. Specifically, MycoCocktail suspensions were dispensed into Mountop and SteriPEN water bottles and UV treated as per the manufacturer instructions once daily for 7 days, followed by a once weekly treatment for up to 56 days. After 4 days of daily UV treatment, both systems achieved a >4 log reduction in MycoCocktail CFU. After the 56-day evaluation period, suspension and biofilm-associated CFU were measured, and a >4 log reduction in CFU was maintained in both systems. Taken together, physical disinfection methods significantly reduced NTM numbers from tap water and may be easy-to-use, accessible applications to reduce environmental NTM exposures from drinking water.

## Introduction

There are an estimated 180,000 individuals living in the United States with nontuberculous mycobacterial (NTM) pulmonary disease (1). A proportion of individuals are at heightened risk for NTM infection and development of pulmonary disease. These include individuals with prior occupational lung damage (e.g., black lung, smoking, and COPD), bronchiectasis, prior infection with *Mycobacterium tuberculosis*, immunodeficiency due to HIV infection, and immunodeficiency due to cancer or chemotherapies (2, 3). In addition, otherwise healthy elderly, slender, and taller women and men are at increased risk for NTM infection (4–6). Individuals in each of these risk groups are also at risk for repeated NTM infections due to their ubiquity in the environment even following the resolution of symptoms after successful antibiotic therapy (7).

NTM are intimately associated with natural and built freshwater systems (8, 9) and linked to exposure from household showerhead water and biofilms by DNA fingerprint comparisons (10). Moreover, NTM and other opportunistic premise plumbing pathogens (OPPPs), including *Pseudomonas aeruginosa, Acinetobacter baumannii, Stenotrophomonas maltophilia*, and *Legionella pneumophila*, surround humans as colonists of drinking water systems (11). As relapse due to NTM reinfection or reactivation of latent infection occurs at frequencies between 25 and 50%, it is of value to identify methods to reduce environmental exposure to NTM in the built household environment.

Reduction of exposure to waterborne NTM could be potentially accomplished by the removal of NTM from household water using a variety of methods, including filtration. To be effective, filtration must prevent the passage of NTM cells, demanding a filter with a pore size of 0.22 μm. These particular filters have been found to reduce the frequency of false-positive acid-fast stains in a tuberculosis laboratory (12) and healthcare-associated infections in bone marrow transplant recipients (13). The replacement of showerheads with 0.22 μm pore size filters also reduced total bacterial counts in shower water and aerosols compared to unfiltered water (14).

In contrast to the 0.22-μm filters, granular activated charcoal (carbon, GAC) filters do not prevent the passage of NTM as their pores are larger than 0.45 μm in diameter (15). In fact, NTM have been shown to colonize GAC filters (15). Specifically, filters loaded with a total of 2.9 × 10^5^ CFU of *Mycobacterium avium* yielded a total of 4.4 × 10^7^ after 3 weeks and 9.4 × 10^7^ CFU after 5 weeks. Although the number of *M. avium* declined after the fifth week, the data showed that the CFU of *M. avium* in the filtrates were far in excess of the number added to the filter, suggesting that the *M. avium* cells can also grow on the filter. Other studies have also demonstrated that passage of rural groundwater through powdered activated carbon filters led to increased numbers of heterotrophic-plate-count bacteria (16).

Although filtration can be used to remove NTM from drinking water, NTM are staggeringly resistant to chlorine and other disinfectants used in drinking water treatment systems. In fact, NTM are substantially more resistant to common methods of water disinfection, such as chlorine, monochloramine, and ozone, than *Escherichia coli* (17). Due to their innate resistance to disinfectants, propensity to form biofilms within piping systems, and ability to survive in low-carbon environments, eradication of NTM from premise plumbing is a nearly impossible task. However, similar to other bacteria, NTM are susceptible to ultraviolet (UV) light (18, 19). Short-wave UV-C radiation (253.7 nm) has been demonstrated to cause lethal damage to microbial biomolecules, most notably by inducing cross-linking of thymine residues in DNA, and is considered an effective germicidal (20). Because of this, a number of large-scale and point-of-use UV water disinfection systems have been developed and are available to consumers (21). However, it has been demonstrated that clinically relevant NTM isolates, such as *M. avium hominissuis*, are more resistant to UV radiation than the coliforms commonly used in water purification system testing (22). Therefore, it is unknown if these commercially available UV-C water disinfection systems could be an effective means of reducing NTM in drinking water.

Herein, we describe the evaluation of the efficacy of filter removal or UV-C–mediated killing of tap water–associated NTM by devices that can be commercially purchased and used by individuals.

## Materials and Methods

### NTM Isolates

*Mycobacterium smegmatis* VT307; *Mycobacterium avium* A5; *Mycobacterium chimaera* MA3820; *Mycobacterium abscessus* P-1-Ay-1; and clinical respiratory isolates *M. abscessus* P2A, *M. avium* P1A, and *M. chimaera* AH16 (23) were used in this study.

### Growth of NTM in Culture

NTM isolates were grown in 50 mL of Middlebrook 7H9 broth (BD, Sparks, MD) containing 0.5% (vol/vol) glycerol and 10% (vol/vol) oleic acid-albumin with aeration (120 rpm) for 4 days at 37°C for *M. smegmatis* and *M. abscessus* or 7 days at 37°C for *M. avium* and *M. chimaera*. In some cases, 500-mL nephelometry flasks were used to measure and record turbidity as absorbance (540 nm) daily and/or cultures were diluted and plated on Middlebrook 7H10 agar plates (BD, Sparks, MD) containing 0.5% (vol/vol) glycerol and 10% (vol/vol) oleic acid-albumin to determine colony forming units (CFU).

### NTM Acclimation to Tap Water

Following growth in microbiological culture media, isolates were acclimated in sterile tap water. Water acclimation was performed in order to replicate the environment within household plumbing based on previously reported differences in disinfectant susceptibility between water- and media-grown NTM isolates (17). Cultures were transferred to sterile 50-mL centrifuge tubes, centrifuged at 5,000 × g for 20 min, supernatants removed, and the cell pellets resuspended in 50 mL of sterile Blacksburg, VA, or Denver, CO, tap water. Centrifugation was repeated, and the cells were again resuspended in 50 mL of sterile Blacksburg or Denver tap water. For water acclimation, suspensions were transferred into sterile 250-mL baffled flasks and incubated at room temperature (filter experiments) or 37°C (UV experiments) with aeration (120 rpm) for 7 days.

### Measurement of Filtration Efficacy of Pall Medical In-line Filters

Suspensions of sterile tap water and *M. smegmatis* were prepared as described above section. Pall-Aqua safe water filters AQ14F1SA and AQ7F1SA 0.2 μm pore size intended for 7- and 14-day use, respectively, were tested (Supplementary Figure 1). Both had void volumes of 33.5 mL, defined as the volume of sterile, disinfectant-free Blacksburg tap water held by the filter before the appearance of any eluate. Pall filters were inoculated by vacuum/pressure-free passage of water-acclimated suspensions of *M. smegmatis* VT307. The number of NTM passing through the filter (i.e., eluate) was measured as CFU on Middlebrook 7H10 agar medium over time. Over the course of the 30-day test period, filters were incubated in an upright position at ambient room temperature (25°C). Samples of filter material were also obtained following the 30-day test period and suspended in sterile tap water, and after vortexing, NTM numbers were measured as CFU on 7H10 agar.

### Passage and Growth of NTM in Granular Activated Carbon (GAC) Filters

A GAC filter system was tested (GE, Smart Water™ Shower Filter, Model GXSM01HWW, GE, Louisville, KY) as depicted in Supplementary Figure 1. The density of each water-acclimated suspension of *M. avium* A5 or *M. abscessus* P-1-Ay-1 in sterile, disinfectant-free Blacksburg tap water was 3 log cells/mL. Two identical filters were inoculated with the volume of cell suspension equal to the void volume and incubated overnight at room temperature. For one of the filters, 1 L of sterile, disinfectant-free Blacksburg tap water was passed through the filter and the eluate collected in a sterile 2-L flask. For the second filter, 1 L of non-sterile, disinfectant-free Blacksburg tap water was passed through the filter and the eluate collected in a second sterile 2-L flask. The bacterial cells in a 50-mL aliquot of each eluate were pelleted by centrifugation (5,000 x g for 20 min), suspended 10 mL sterile disinfectant-free tap water, and the CFU/mL of the concentrated suspension measured by spreading 0.1 mL of each suspension on 7H10 agar. The effect of passage of 1 L of sterile, disinfectant-free tap water (Filter 1) or non-sterile, disinfectant-free tap water (Filter 2) through the filters on NTM colony counts was measured weekly for up to 8 weeks.

Next, to measure the CFU on GAC filters, the filter was removed from the filter holder and cut into 1-cm sections using a saw that was sterilized by alcohol flaming. Each section was broken up using a sterilized mortar and pestle, and 1 gm of the material was suspended in 10 mL of sterile 0.01 M Tris buffer (pH 7), 10^6^ M Zwittergent 3–12, 10^3^ M EGTA, and 0.01% peptone (24) to disrupt hydrophilic and hydrophobic associations between NTM and GAC particles, and shaken (one reciprocation per second) for 60 min at room temperature. The GAC particles were allowed to settle by gravity, and the CFU/mL in the supernatant was determined by spreading 0.1 mL of each suspension on 7H10 agar. Colonies were counted after 10–14 days incubation at 37°C. The bacterial density of the GAC particulate suspension and the total NTM CFU that were bound to the 1-cm section of the GAC filter were calculated. The total NTM CFU in the GAC filter was calculated by summing the values for each section. As a control, a GAC filter was inoculated as described above and immediately removed and processed to measure the total NTM per filter to provide counts actually added and recovered from each filter.

Sterile tap water was continuously passed through filters and samples of the eluate were collected immediately after passing the suspension through the filter every 3 days to 4 weeks. At each time point, the number of CFU passing through the filter was measured. After the 30-day use period, filter samples were collected and suspended and CFU per gm of filter measured.

###  “MycoCocktail” Preparation

A schematic detailing the preparation of the “MycoCocktail” is shown in Supplementary Figure 2. In brief, after water acclimation, suspensions were transferred to 50-mL conical vials and vortexed thoroughly. Suspensions were then diluted 1:1,000 in sterile tap water and mixed together in order to achieve a MycoCocktail containing a mixture of *M. abscessus, M. avium*, and *M. chimaera* with a total CFU between 1 x 10^4^ and 1 x 10^5^/mL. MycoCocktail was subsequently used in inoculation of LifeStraw GO, Mountop, and SteriPEN systems and prepared fresh for each independent experiment.

### Measurement of Filtration Efficacy of LifeStraw Go Bottle

The LifeStraw Go (LIFESTRAW S.A., Lausanne, Switzerland) bottle contained two filters: an activated carbon capsule and a hollow fiber membrane (Supplementary Figure 3). On first use, LifeStraw Go bottles were rinsed twice with 650-mL volumes of sterile Blacksburg tap water in order to remove any materials remaining from bottle manufacturing or assembly. After two-fold washing, the bottles were filled with 650 mL of sterile Blacksburg tap water and incubated overnight to thoroughly wet the filters. A 650-mL aliquot of MycoCocktail was dispensed into the LifeStraw Go bottle and allowed to equilibrate for 1 day to simulate the typical consumer filling, but not immediately using, the bottle. Using sterile tubing and a sterile suction flask, 100 mL of the liquid was withdrawn into the suction flask (Supplementary Figure 4D). The number of NTM on 7H10 agar was measured. The lid of the LifeStraw Go bottle was unscrewed, and 100 mL of MycoCocktail was added and incubated overnight. Every day after inoculation, sample collection and MycoCocktail enumeration was repeated for an additional 10 days.

### Testing Two UV-C Disinfection Systems Against NTM

Two different types of UV-C disinfection water bottle systems were tested: Mountop Water Purifier Bottle (201330464721.4; GE) and SteriPEN Aqua UV Water Purifier Kit (Article #60110075; Brita LP, Oakland, CA). Both bottle systems utilize a 90-s treatment cycle of 254-nm UV-C radiation to disinfect 750 mL (Mountop) or 1 L (SteriPEN) of drinking water. Differences in UV irradiation between both water bottle systems are depicted in Supplementary Figure 3. Mountop is equipped with a UV bulb on the bottom surface of the cap that emits UV-C radiation directly above the air–liquid interface. SteriPEN Aqua utilizes a cylindrical UV bulb that is completely submerged and is gently stirred during the treatment cycle. For all testing of Mountop and SteriPEN Aqua, three different bottle systems were screened in order to capture potential inconsistencies in the performance of each unit. Prior to evaluation of killing by UV-C radiation, systems were sterilized using Prolystica® enzymatic cleaner (STERIS, Mentor OH) and household bleach diluted in sterile Denver tap water. Water bottles were filled with 4:1,000 Prolystica®:sterile tap water and agitated vigorously on a rocking platform for 5 min. 1:1,000 bleach:sterile tap water was then added, and bottles were returned to shake for 5 min. Solutions were then discarded, and the bottles were filled with sterile tap water and shaken for 5 min to rinse. Rinsing steps were repeated two additional times, and after the final rinse, bottles were allowed to dry overnight at room temperature in a biosafety cabinet.

#### Measurement of the Efficacy of Mountop and SteriPEN Systems

In order to determine the effect of a single UV treatment (Supplementary Figure 4A), each bottle was inoculated with 750 mL (Mountop) or 1 L (SteriPEN) of freshly prepared MycoCocktail; 1 mL of the inoculum was removed immediately after inoculation, serially diluted, and plated for CFU on 7H10 agar to measure the initial CFU in the inoculum. Bottles were immediately sterilized with UV-C radiation according to the manufacturer's instructions (90-s treatment with 254 nm light), and 1 mL of the treated suspension was removed to determine the post-treatment NTM burden. For all UV-treated bottles, untreated bottles were included as controls. Suspensions were spread plated and also independently serially diluted and plated in duplicate for CFU on 7H10 agar. Images were taken of spread plates and, in regions where NTM growth was difficult to visualize, a region of representative growth was chosen and magnified approximately six-fold.

For 7-day evaluation of the efficacy of Mountop and SteriPEN with no water replacement (Supplementary Figure 4B), bottles were inoculated with MycoCocktail as described and incubated overnight at room temperature. The following day, bottles were UV-C irradiated per the manufacturer's instructions. After disinfection, 1 mL of the suspension from each bottle was removed and plated for CFU. These steps were repeated daily for 7 days.

For 56- or 21-day evaluation with water replacement (Supplementary Figure 4C), bottles were incubated overnight at room temperature after MycoCocktail inoculation. The following day, bottles were removed and UV-C irradiated to disinfect the cocktail (25, 26). The suspension in each bottle was then transferred to 1-L, 0.22-μm pore filters (Foxx Life Sciences LLC, Salem NH) and vacuum filtered. Filter membranes were excised using sterile razor blades and forceps and added to 10 mL of sterile Denver tap water in a 50-mL conical vial. Excised filters were vortexed thoroughly for 1 min in order to remove filter-associated NTM and 1 mL of the suspension was serially diluted and plated for CFU as previously described. Bottles were then refilled with sterile tap water and returned to incubate at room temperature. UV treatment, suspension plating, and water replacement were performed daily for 7 days after inoculation and then weekly until 21 or 56 days post-inoculation. All methods used with UV-treated bottles were replicated with untreated bottles with the exception of UV disinfection.

#### Biofilm Sampling of Mountop and SteriPEN Systems

In order to evaluate the contribution of biofilm formation on the inner surface of the water bottles, biofilms in Mountop and SteriPEN bottles were sampled. Four regions of each bottle were sampled: (1) the bottle cap, (2) a 4-cm^2^ region of the air–liquid interface, (3) a 4-cm^2^ region of the middle of the bottle, and (4) a 4-cm^2^ region of the bottom of the bottle using a sterile synthetic swab (see schema detailed in **Figure 4A**). Swabs were collected, vortexed for 1 min in 1 mL sterile Denver tap water, and the suspension plated for CFU.

#### Comparing UV-C Susceptibility of NTM Isolates

After completion of 56 days of UV-disinfection and water replacement, representative colonies of *M. abscessus* P2A, *M. avium* P1A, and *M. chimaera* AH16 were picked from CFU plates. These isolates are referred to as “UV-adapted” and were inoculated into 8 mL Middlebrook 7H9 broth with ADC enrichment to establish stocks. Colonies of water-acclimated *M. abscessus, M. avium*, and *M. chimaera* were also picked, inoculated into 7H9 broth and prepared into stocks. Stocks were stored at −80°C until use. To compare differences in UV susceptibility between the water-acclimated and UV-adapted NTM isolates, 100 μl of each isolate was spread onto 7H10 agar. Spread plates were then exposed to 90 s of 254-nm UV-C radiation. During exposure, one half of the agar plate was covered with a notecard, and the other half remained directly exposed to UV-C. Plates were incubated at 37°C until growth appeared, and UV-C susceptibility of each isolate was determined qualitatively by the difference in growth between the covered and uncovered portion of the agar plate.

### Statistics

Graphpad Prism 8 was used to perform unpaired *t-*tests as a measure of statistical significance. For all data shown, *p* < 0.05 was considered statistically significant. For multiple *t-*tests, *P*-values were adjusted using the Holm–Sidak method. Data shown are mean ± *SEM* for two or more independent experiments.

## Results

### Efficient Removal of NTM by Pall Medical In-line Filters

Immediately and daily for 13 (7-day filter) or 25 days (14-day filter), 100 mL of the *M. smegmatis* tap water suspension was added through the input line of the filter, and the liquid flowing out (~100 mL) was collected in a sterile flask. As triplicate samples were spread from the 100 mL effluent, the limit of detection was <3.3 CFU/mL. Although *M. smegmatis* was recovered from the swabbed surface samples of the input side of the 7- and 14-day filters (77 and 57 CFU/cm^2^, respectively), all effluent water passing through either the 7- or 14-day Pall filters over the 13- or 25-day test period were completely devoid of *M. smegmatis* (data not shown).

### Inefficient Removal of NTM by GAC Filters

Although water filtered through a GAC filter modestly reduced *M. abscessus* and *M. avium* numbers in effluent water (Tables 1A, 2A, respectively), removal was inconsistent and varied in count from below the limit of detection to over 4 log *M. abscessus* and *M. avium* per milliliter of effluent water. NTM were not only recovered from filter effluents across the 30-day sampling period, but viable NTM were also recovered from the GAC filters (Tables 1B, 2B). Filter-associated burden of *M. abscessus* was much less than *M. avium* with, on average, ~53 CFU/gm (*M. abscessus*) and ~482 CFU/gm (*M. avium*) recovered from filter sections.

**Table 1A:** *M. abscessus* in effluent water from GAC filter.

**Time (days)**	***M. abscessus*** **CFU/mL**	
	**Unfiltered**	**Filtered**
0	8.3 × 10^5^	<3.3 × 10^2^
1	3.2 × 10^5^	7.0 × 10^3^
2	4.0 × 10^5^	<3.3 × 10^2^
3	1.6 × 10^6^	<3.3 × 10^2^
4	6.6 × 10^4^	3.0 × 10^4^
5	2.4 × 10^4^	3.3 × 10^3^
6	5.1 × 10^6^	<3.3 × 10^2^
7	1.1 × 10^5^	<3.3 × 10^2^
8	3.1 × 10^5^	6.7 × 10^3^

Inoculum = 1.5 × 10^5^ CFU.

**Table 2A:** *M. avium* in effluent water from GAC filter.

**Time (days)**	***M. avium* CFU/mL effluent water**	
	**Unfiltered**	**Filtered**
0	1.0 × 10^6^	1.0 × 10^4^
1	3.0 × 10^5^	<3.3 × 10^3^
2	6.5 × 10^4^	<3.3 × 10^3^
3	1.1 × 10^6^	<3.3 × 10^3^
4	1.8 × 10^5^	<3.3 × 10^3^
5	4.0 × 10^5^	1.0 × 10^4^
6	6.0 × 10^5^	<3.3 × 10^3^
7	6.9 × 10^6^	<3.3 × 10^3^
8	1.5 × 10^6^	4.7 × 10^4^

Inoculum = 2.0 × 10^5^ CFU.

**Table 1B:** *M. abscessus* in GAC filter.

**Filter sample**	**CFU/gm**
	**GAC**
FC-1 (Top)	48/gm
FC-2	54/gm
FC-3	<8/gm
FC-4	48/gm
FC-5	18/gm
FC-6 (Bottom)	145/gm

Inoculum = 1.5 × 10^5^ CFU.

FC, “fractions cut” (6 – 1cm fractions total).

**Table 2B:** *M. avium* in GAC filter.

**Filter sample**	**CFU/gm**
	**GAC**
FC-1 (TOP)	1,100/g
FC-2	300/g
FC-3	500/g
FC-4	330/g
FC-5	430/g
FC-6 (Bottom)	230/g

Inoculum = 2.0 × 10^5^ CFU.

*FC, “fractions cut” (6 – 1cm fractions total)*.

### Total Removal of NTM by LifeStraw Go Bottle

MycoCocktail contained, on average, 5.7652 log *M. avium* A5, *M. chimaera* MA3820, and *M. abscessus* P-1-Ay-1 cells/mL at inoculation, a 1 x 10^4^- to 1 x 10^5^-fold higher concentration than typical culturable NTM burden in drinking water (27, 28). Over the course of 68 days, 100 mL of water was periodically removed aseptically through the drinking tube by suction and the number of NTM counted. Assuming that the bottle would be refilled due to consumption, the 100 mL sampled was replaced by 100 mL of MycoCocktail. At every sampling point (1, 4, 5, 6, 7, 11, 18, 25, 32, 40, 46, 53, and 68 days post-inoculation), no NTM (<3.3 CFU/mL) were recovered (data not shown).

#### Efficient Removal of NTM by Both Mountop and SteriPEN Systems After a Single UV Treatment

**Figure 1:**
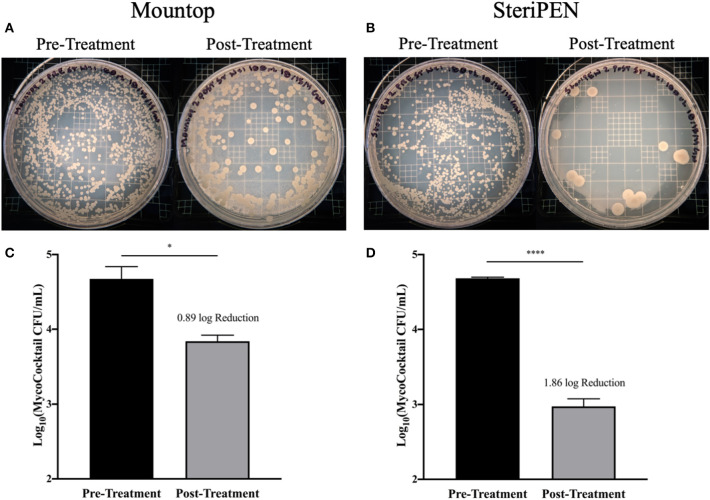
NTM CFU is significantly reduced after single UV treatment in both and Mountop and SteriPEN systems. Mountop **(A,C)** and SteriPEN **(B,D)** systems were inoculated with MycoCocktail and immediately UV-C disinfected per the manufacturer's protocol. **(A,B)** Representative images of MycoCocktail CFU in Mountop and SteriPEN before and after UV treatment. **(C,D)** Log(MycoCocktail CFU) before (pretreatment) and after (post-treatment) UV disinfection. *n* = 3 independent CFU measurements per time point. ns, not significant; significant at *p < 0.05, ****p < 0.0001.

The efficiency of the manufacturer's standard protocol for the use of Mountop and SteriPEN systems in the reduction of MycoCocktail CFU in drinking water was evaluated (see schema, Supplementary Figure 4A). For both Mountop and SteriPEN systems, a single UV treatment significantly reduced the initial bacterial burden within each bottle (Figure 1). A stark reduction in inoculum CFU was observed on 7H10 agar plates spread with MycoCocktail pre- and post-treatment with UV-C in both systems (Figures 1A,B). However, as illustrated by the agar plate images, the reduction in bacterial burden achieved with the Mountop system was less than with SteriPEN. In the Mountop system, the reduction in NTM burden was, on average, 0.8850 log (4.7404 log to 3.8553 log; *p* < 0.05; Figure 1C), and the SteriPEN system achieved an average reduction of 1.8603 log (4.6842 log to 2.8240 log; p < 0.0001; Figure 1D) after a single UV treatment.

#### Mountop Is Less Effective Than SteriPEN in Disinfecting NTM From Tap Water When Used Consistently for 7 Days

**Figure 2:**
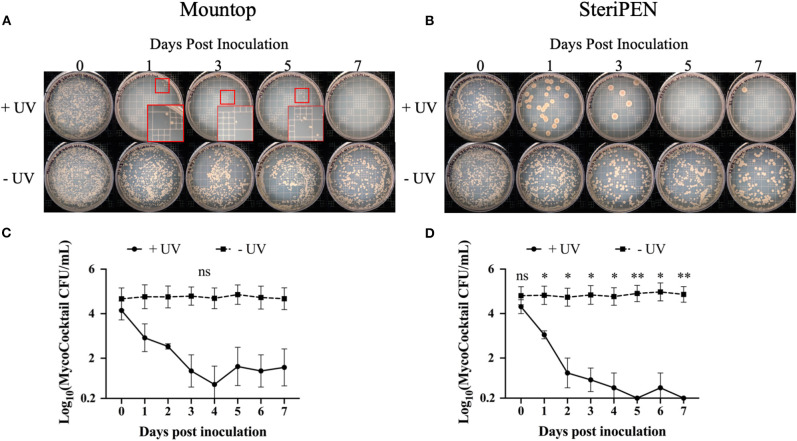
NTM CFU is reduced by UV-C treatment. Mountop **(A,C)** and SteriPEN **(B,D)** systems were inoculated with MycoCocktail and allowed to incubate at room temperature overnight. After incubation, the MycoCocktail was UV-C disinfected per the manufacturer's protocol. “+UV” = water that was UV treated. “-UV” = water that was not exposed to UV. One milliliter of suspension was removed and plated for CFU and the systems were returned to incubate at room temperature. CFU plating was repeated daily for 7 days post-inoculation. **(A,B)** Representative images of MycoCocktail CFU in Mountop and SteriPEN across 7 days of daily UV-C disinfection. Smaller red boxes indicate regions where growth was difficult to visualize. Larger red boxes show colonies magnified ~6-fold. **(C,D)** Log(MycoCocktail CFU) in SteriPEN and Mountop systems. *n* = 3 independent CFU measurements per timepoint. ns, not significant; significant at *p < 0.05, **p < 0.01.

Next, the change in CFU after a single, initial inoculation of MycoCocktail in the presence or absence of UV-C treatment across a 7-day period (see schema, Supplementary Figure 4B) was evaluated. For the Mountop and SteriPEN bottles that were not UV-C treated, CFU remained consistent across 7 days (Figures 2A,B bottom row). However, with daily UV-C treatment, MycoCocktail CFU was progressively reduced across the sampling period as deduced by visual observation of viable colonies on 7H10 agar plates (Figures 2A,B top row). For the untreated MycoCocktail, NTM burden from inoculation to the end of the 7-day interval was 5.0544 log to 5.0669 log for SteriPEN and 5.0072 log to 5.0000 log for Mountop (Figures 2C,D). In comparing these values to UV-C treated MycoCocktail, the differences between treated and untreated bottles was not statistically significant for the Mountop system (4.5662 log to 2.3680 log across 7 days; Figure 2C). However, a significant decline in CFU was observed for the SteriPEN water bottle system compared to the untreated control (~4.5441 log reduction across 7 days; Figure 2D).

#### Simply Replacing the Water in Mountop and SteriPEN Systems Reduced NTM Similar to UV-C Treatment

**Figure 3:**
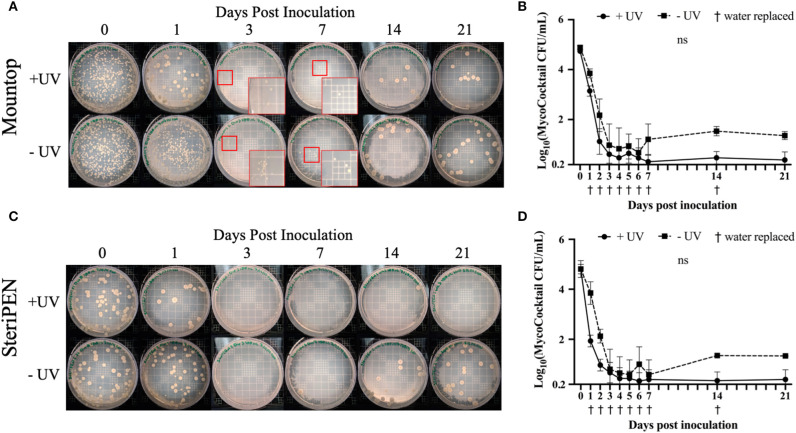
The simple action of water replacement reduces NTM counts. Mountop **(A,B)** and SteriPEN **(C,D)** systems were inoculated with MycoCocktail and allowed to incubate at room temperature overnight. After incubation, the MycoCocktail was UV-C disinfected as per the manufacturer's protocol (+UV) or not exposed to UV (–UV). The suspensions within each system were filtered, plated for CFU, and the suspension replaced with sterile tap H_2_O. CFU plating and water replacement was performed daily for 7 days and weekly up to 21 days post-inoculation. **(A,C)** Representative images of MycoCocktail CFU in Mountop and SteriPEN across 21 days of UV-disinfection. Smaller red boxes indicate regions where growth was difficult to visualize. Larger red boxes show colonies magnified ~6-fold. **(B,D)** Log(MycoCocktail CFU) across 21 days with and without UV treatment by Mountop or SteriPEN. *n* = 3 (+UV) or *n* = 2 (–UV) independent CFU measurements per timepoint. ns, not significant.

To determine the effect of water replacement on NTM CFU, both water bottle systems were initially inoculated with 4.8–4.9 log MycoCocktail CFU, UV-C irradiated, and water was replaced with sterile tap water daily for 7 days and weekly up until 21 days post-inoculation (see schema, Supplementary Figure 4C). Across all time points, CFU decreased for both Mountop and SteriPEN. This observation is illustrated by the similar appearance of NTM colony growth on 7H10 agar plates regardless of UV treatment (Figures 3A,C). Although the comparable decline in inoculum CFU in the absence of UV-C treatment was unexpected, unlike the UV-C treated inocula, the NTM burden in the untreated Mountop and SteriPEN bottles increased to ~1 log/L at 14 and 21 days post-inoculation. For the UV-treated Mountop system, the average decline in CFU after the first UV treatment was 1.5950 log, and although this decline continued over time, it failed to consistently reach 0 CFU/mL across the 21-day observation interval (Figure 3B). The UV-treated SteriPEN achieved, on average, a 2.8744 log reduction in NTM counts after a single UV-C treatment and continued to decline to 0.3920 log CFU/mL by 4 days post-inoculation (Figure 3D).

### Biofilm CFU Within Mountop and SteriPEN Bottles Is Negligible

**Figure 4:**
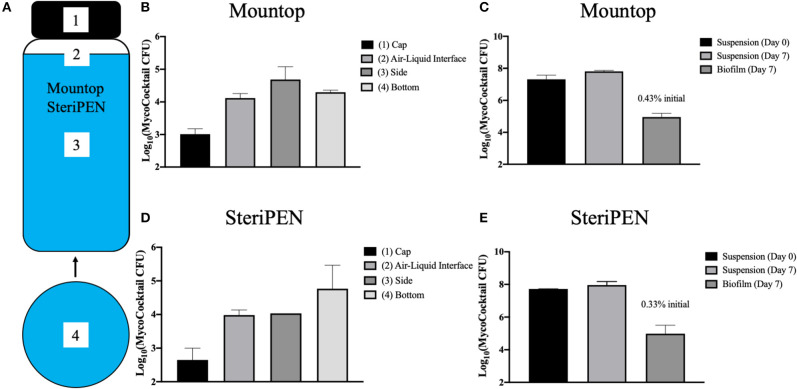
Biofilm formation within Mountop and SteriPEN water bottles contribute little to total CFU. **(A)** Four regions of each water bottle system were sampled with sterile synthetic swabs to quantify biofilm-associated CFU: the interior surface of the bottle cap (1), a 4-cm^2^ region at the air–liquid interface (2), a 4-cm^2^ region completely submerged in inoculum (3), and a 4-cm^2^ region of the interior bottom surface of the bottle (4). Mountop **(B,C)** and SteriPEN **(D,E)** systems were inoculated with MycoCocktail and an aliquot of the Day 0 suspension was plated for CFU. Bottles were then allowed to incubate undisturbed at room temperature for 7 days. At Day 7, an aliquot of the suspension was plated for CFU and biofilms within each system were sampled. Biofilm swabs were then plated for CFU and the CFU/cm^2^ in each area was applied to each bottle's total surface area. Biofilm CFU was calculated as a percentage of the initial Day 0 suspension. **(B,D)** CFU of each region sampled for biofilm-associated CFU. **(C,E)** CFU of suspensions at Day 0 and Day 7 are compared to that of the biofilm at day 7. *n* = 2 independent experiments.

Due to the hydrophobicity of NTM, the inner sections of each bottle were evaluated for the formation of NTM biofilms. Specifically, after 7 days of incubation with MycoCocktail, the bottles were emptied and the NTM CFU remaining in suspension enumerated. Next, four different areas of the emptied bottles were sampled for biofilms in the cap, air–liquid interface, side, and bottom wall (Figure 4A). In both Mountop and SteriPEN bottle systems, the cap contained the lowest number of detectable CFU and the side of the Mountop bottle and the bottom surface of the SteriPEN bottle showed the highest biofilm-associated CFU (Figures 4B,D). Although the CFU in suspension did not significantly differ between Day 0 and Day 7, biofilm associated CFU in both Mountop and SteriPEN accounted for <1% of the total CFU (Figures 4C,E).

### UV-C Exposed NTM Do Not Show Increased Resistance to UV

**Figure 5:**
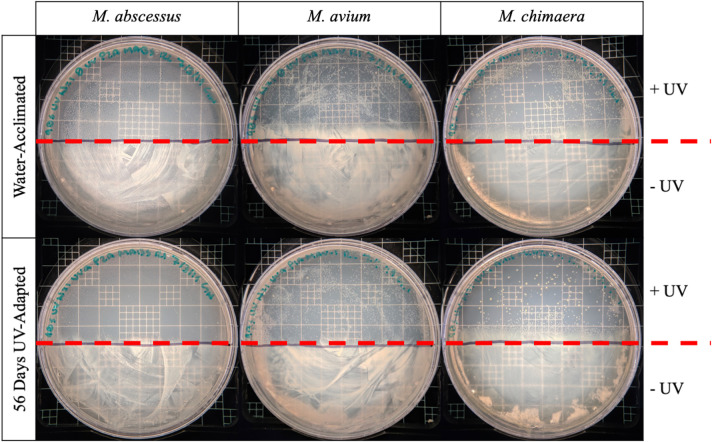
Repeated UV exposure does not affect the susceptibility of NTM to UV-C. The sensitivity to UV-C treatment between unexposed (water acclimated, top row) *M. abscessus, M. avium*, and *M. chimaera* and 56-day UV-adapted *M. abscessus, M. avium*, and *M. chimaera* (56-days UV-adapted, bottom row) were compared for their ability to survive 90 s of UV-C irradiation. One hundred microliter of bacterial stock was spread onto 7H10 agar plates. Half of the plate was covered with by notecard to protect against UV-exposure and the other half was exposed directly to 254-nm UV-C light for 90 s. Plates were incubated at 37°C and the growth of the UV-C treated (above red line) and untreated (below red line) regions were visually compared.

Finally, we tested the possibility that the NTM that survived UV-C treatment after 56 days of incubation were innately resistant to UV-C irradiation. However, no significant difference in the growth of water-acclimated NTM and UV-C adapted isolates were observed (Figure 5). In comparing the UV-susceptibility between isolates of different species, it was observed that *M. abscessus* was most susceptible to UV-C treatment (100% killed in UV treatment), whereas *M. avium* and *M. chimaera* were less affected by UV-C treatment (Figure 5).

## Discussion

Methods to reduce environmental NTM exposures are critically needed. Because there is potential for NTM in tap water to be ingested and refluxed to cause lung infections (29, 30), suggestions to reduce bacterial numbers in drinking water are particularly important and timely. In this study, we tested a variety of physical methodologies to reduce NTM and demonstrate that both water filtration using Pall filters (section Efficient Removal of NTM by Pall Medical In-Line Filters) and UV-C irradiation (Figures 1–4) are effective in reducing the number of *M. smegmatis* (Pall filter) as well as *M. abscessus, M. avium*, and *M. chimaera* (UV-C) from tap water.

In this work, we show that GAC filters do not prevent the passage of NTM and actually support the growth of NTM within the filter (Tables 1, 2), results similar to other studies (15). However, in the case of point-of-use Pall filters and LifeStraw Go systems with ≤ 0.2 μm pore size, the reduction in CFU were at the limit of detection of NTM colonies, yet we refrain from making any wide-sweeping recommendations on whether whole residence or point-of-use filtration is more effective. Because it is likely that the majority of NTM in premise plumbing are resident in biofilms, whole-house filtration at the entry point for water would not theoretically be of use as the filtered water would be inoculated from the biofilm. However, we believe that point-of-use filtration at taps and showerheads would prove more effective in reducing exposure. It must be acknowledged that point-of-use filters are expensive and have a defined, manufacturer-indicated use life, after which they can break down or clog. To prevent clogging and extend its useful life, a 1- to 5-μm pore size prefilter can be employed to intercept larger particulates before they clog smaller pore size filters.

In assessments of Mountop and SteriPEN personal UV-C disinfection water bottle systems, we found that both systems were effective in eliminating *M. abscessus, M. avium*, and *M. chimaera* in a MycoCocktail (Figure 1) when the manufacturer's recommendations are followed. We also found that SteriPEN was more effective than Mountop in reducing NTM counts in water held in the bottle for 7 days (Figure 2). In the 21-day evaluation of suspension CFU with water replacement, a >3 log reduction in NTM CFU is observed both with and without UV treatment in either system (Figure 3). Because biofilm formation was found to contribute <1% of the total CFU contained within each bottle (Figure 4), it can be concluded that water replacement was responsible for the elimination of inoculum CFU in the absence of UV treatment. In contrast, when the water is not replaced regularly, the CFU remains rather stable across 7 days in the absence of UV and UV-C treatment reduces NTM counts (Figure 2) across a 7-day interval. This finding is corroborated by the observed decline in inoculum CFU after just a single UV treatment, in which the Mountop system eliminated 0.8850 log NTM CFU and SteriPEN eliminated 1.8603 log NTM CFU (Figure 1). The reduced disinfection efficacy of the Mountop bottle may be due to the innate architecture of the water bottle systems. In the Mountop system, UV is emitted from the bottle cap, whereas in the SteriPEN, the UV lamp is submerged in the water from the top to almost the bottom of the bottle. Therefore, as UV dose is a function of distance, cells are likely exposed to a higher UV dose in the SteriPEN. Further, as UV must cross the air–liquid interface in the Mountop, it could be refracted, thus reducing dose (schema, Supplementary Figure 3). Although further analyses are needed to definitively determine why these two methods of UV treatment differ in their capacity to kill NTM, these findings suggest that the differences in the design of point-of-use UV-disinfection systems are important factors in their bactericidal activity. Thus, factors such as frequency of regular water replacement and the method of UV-C irradiation used likely contribute to NTM survival in these systems.

For UV-C irradiation to be effective, particulates should be removed by installation of a 1- to 5-μm pore size filter upstream of the irradiation unit and the clarity of the UV-irradiation chamber maintained by regular cleaning. Although the advantages of UV-disinfection for large- and small-scale applications is well-established, there is no information available to judge whether the dosages of UV are high enough in the bottles to increase the frequency of viable mutants; an unlikely but possible contraindication for its use. For example, would a consequence of UV disinfection in the bottles lead to the selection of UV-resistant mutants? Our data suggest that UV-C exposure does not lead to increased selection or production of UV-resistant NTM (Figure 5); however, more robust, large-scale analyses of the mutagenic consequences of repeated UV exposure on waterborne NTM are needed in future studies. In addition, although we found minimal biofilm formation on the inner surfaces of the water bottles after 7 days of incubation with MycoCocktail (Figure 4), the possibility of NTM-biofilm formation on unclean surfaces or in inadequately cleaned water bottles is likely to increase the resistance of tap water–associated NTM to disinfection by, potentially, a 100-fold (31).

A limitation of these studies is that NTM dose experiments were not performed, and conclusions are based on the change in CFU over time compared to the number of the initial inoculum and physical disinfection method used. However, as different species and isolates of NTM were tested, we predict that the results would be similar for other NTM species when testing these particular disinfection methods. In addition, although UV-C irradiation and filtration were independently assessed for their ability to reduce waterborne NTM burden, the compounding effect of both methods was not investigated in this study. Because both methods of water disinfection produced significant reductions in NTM CFU, we predict that the combination of methods would produce even more effective removal of NTM from drinking water. Furthermore, in order to be directly applicable to consumers, water treatment was only performed as designated by the manufacturers' protocols or typical use, and alternative treatment strategies that could have more significant impacts on NTM CFU were not investigated. Similarly, variables affecting typical consumer usage of these systems (i.e., water agitation, detergent-based cleaning, water storage container) were not manipulated in production of these data. The effect of UV and filter-based methods of water disinfection were performed using sterile tap water from Blacksburg, VA, or Denver, CO. Although the methods of disinfection assayed in this study worked equally well regardless of the tap water source, it is possible that variation in the chemistries of the tap water used in this study (i.e., pH, salinity, hardness) could have impacted the performance of the disinfection systems. Although beyond the scope of this study, it would be prudent to also test these methods of disinfection on the removal of NTM from natural waters, such as freshwater streams, where turbidity and leaf-litter likely impact the effectiveness of filtration or UV-disinfection (32).

Our results implicate the usefulness of filtration and UV-disinfection methodologies on the removal of NTM from U.S. municipal drinking water systems. However, in developing nations where access to municipal water may be limited, natural surface, and groundwater utilized for human consumption may also serve as important sources of NTM exposure. In rural West African communities and the Western Pacific, natural sources of drinking water, often of low quality, have been associated with *Mycobacterium ulcerans*, the causative agent of Buruli skin ulcers (33–36). However, the prevalence of pulmonary-associated NTM, such as MAC and *M. abscessus*, have not yet been extensively studied in respiratory specimens or in drinking water, which may be complicated by the high prevalence rates of *Mycobacterium tuberculosis* infection in certain low-income countries. Nonetheless, it would be useful to test the disinfection methods examined in this study for their effectiveness in removing NTM in drinking water from low-income countries globally and compare these results to drinking water from other developed countries where NTM pulmonary disease has been extensively studied (37–40).

Among the physical methods of water disinfection screened, both filtration and UV-C disinfection proved effective in significantly reducing the viable NTM population in spiked tap water. Based on these findings, we propose that commercially available water purification systems could provide a simple way for individuals at risk for NTM infection to reduce their exposure to waterborne NTM. Although further study is needed, these data are the first to address and suggest promise for point-of-use water purification devices against clinically relevant NTM species that reside in municipal drinking water systems of man-made built environments.

## Data Availability Statement

All datasets generated for this study are included in the article/Supplementary Material.

## Ethics Statement

Clinical NTM isolates were from de-identified patients and ethical approval was reviewed by the National Jewish Health, Kaiser Permanente Hawai'i, and University of Colorado Institutional Review Boards (IRB) under protocols #HS-2903, FWA #00002344, and #15-0511, respectively, and determined the requirement for informed consent were waived.

## Author Contributions

JF and JH conceived and supervised the project. GN and MW performed the experiments. GN and JF performed statistical analyses. GN, JF, and JH wrote and edited all versions and the final manuscript.

## Conflict of Interest

The authors declare that the research was conducted in the absence of any commercial or financial relationships that could be construed as a potential conflict of interest.
