# Supplementary material for: Physical Measures to Reduce Exposure to Tap Water–Associated Nontuberculous Mycobacteria
Source: Front Public Health. 2020 Jun 12;8:190. doi: 10.3389/fpubh.2020.00190 (PMC7304319; doi:10.3389/fpubh.2020.00190)
Supplement: Supplementary file 1 [file Presentation_1.pptx]

## Slide 1
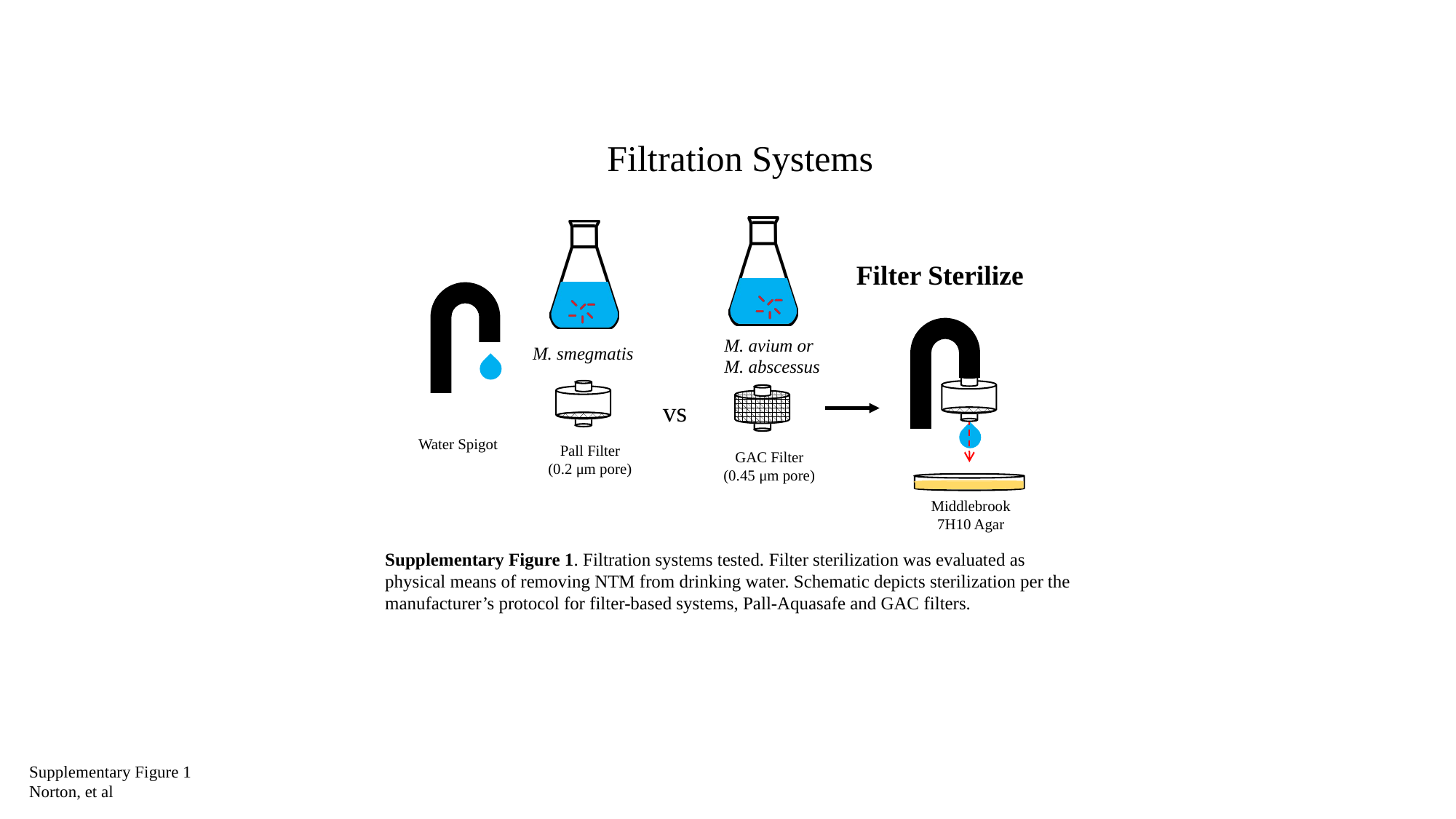

Filtration Systems
Filter Sterilize
M. avium or M. abscessus
M. smegmatis
vs
Water Spigot
Pall Filter
(0.2 μm pore)
GAC Filter
(0.45 μm pore)
Middlebrook
7H10 Agar
Supplementary Figure 1. Filtration systems tested. Filter sterilization was evaluated as physical means of removing NTM from drinking water. Schematic depicts sterilization per the manufacturer’s protocol for filter-based systems, Pall-Aquasafe and GAC filters.
Supplementary Figure 1
Norton, et al

## Slide 2
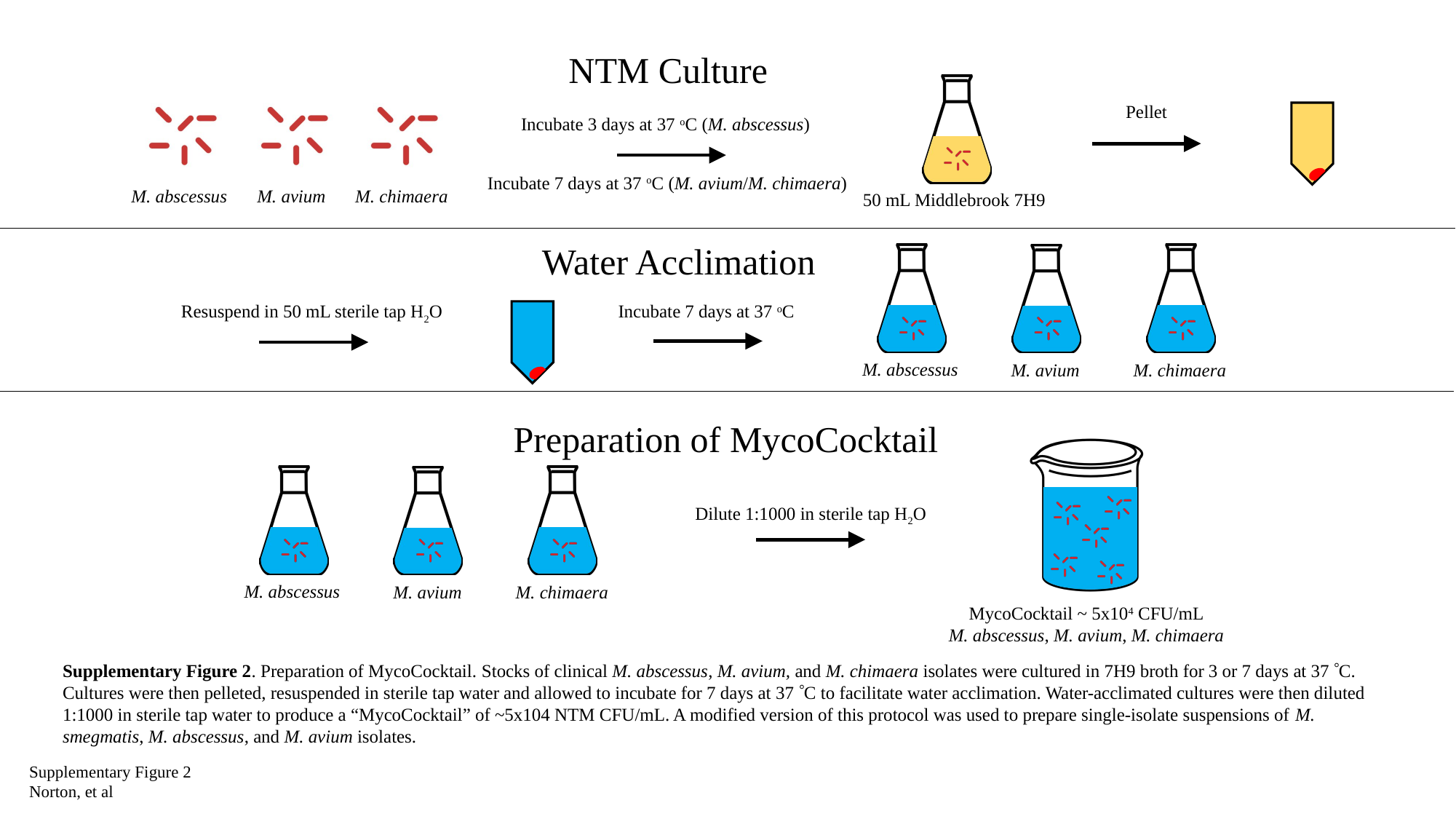

NTM Culture
50 mL Middlebrook 7H9
Pellet
M. abscessus
M. avium
M. chimaera
Incubate 3 days at 37 oC (M. abscessus)
Incubate 7 days at 37 oC (M. avium/M. chimaera)
Water Acclimation
M. abscessus
M. avium
M. chimaera
Resuspend in 50 mL sterile tap H2O
Incubate 7 days at 37 oC
Preparation of MycoCocktail
Dilute 1:1000 in sterile tap H2O
M. abscessus
M. avium
M. chimaera
MycoCocktail ~ 5x10^4^ CFU/mL
M. abscessus, M. avium, M. chimaera
Supplementary Figure 2. Preparation of MycoCocktail. Stocks of clinical M. abscessus, M. avium, and M. chimaera isolates were cultured in 7H9 broth for 3 or 7 days at 37 °C. Cultures were then pelleted, resuspended in sterile tap water and allowed to incubate for 7 days at 37 °C to facilitate water acclimation. Water-acclimated cultures were then diluted 1:1000 in sterile tap water to produce a “MycoCocktail” of ~5x10^4^ NTM CFU/mL. A modified version of this protocol was used to prepare single-isolate suspensions of M. smegmatis, M. abscessus, and M. avium isolates.
Supplementary Figure 2
Norton, et al

## Slide 3
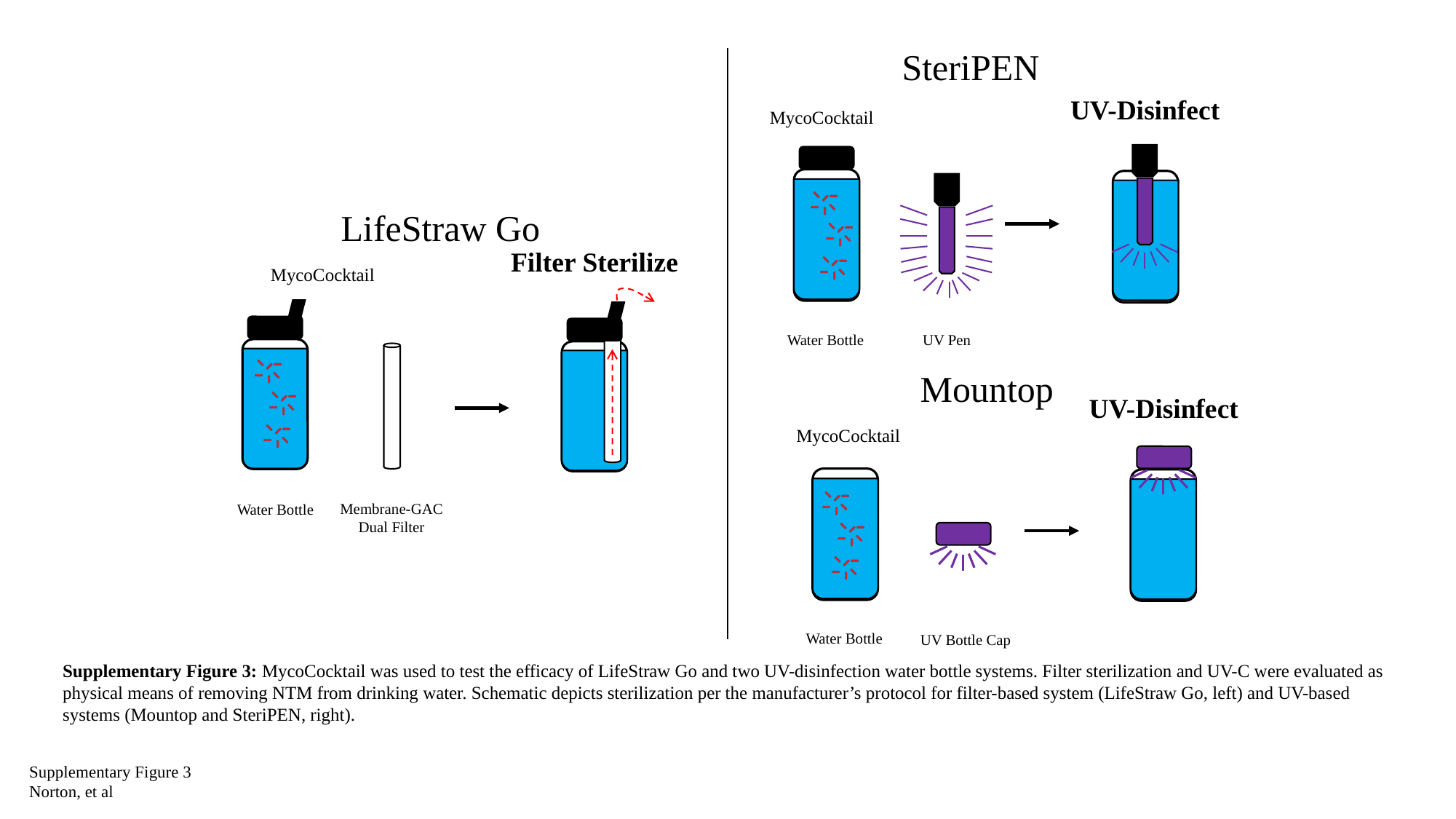

SteriPEN
UV-Disinfect
Water Bottle
UV Pen
MycoCocktail
LifeStraw Go
Filter Sterilize
Membrane-GAC
Dual Filter
Water Bottle
MycoCocktail
Mountop
UV-Disinfect
Water Bottle
UV Bottle Cap
MycoCocktail
Supplementary Figure 3: MycoCocktail was used to test the efficacy of LifeStraw Go and two UV-disinfection water bottle systems. Filter sterilization and UV-C were evaluated as physical means of removing NTM from drinking water. Schematic depicts sterilization per the manufacturer’s protocol for filter-based system (LifeStraw Go, left) and UV-based systems (Mountop and SteriPEN, right).
Supplementary Figure 3
Norton, et al

## Slide 4
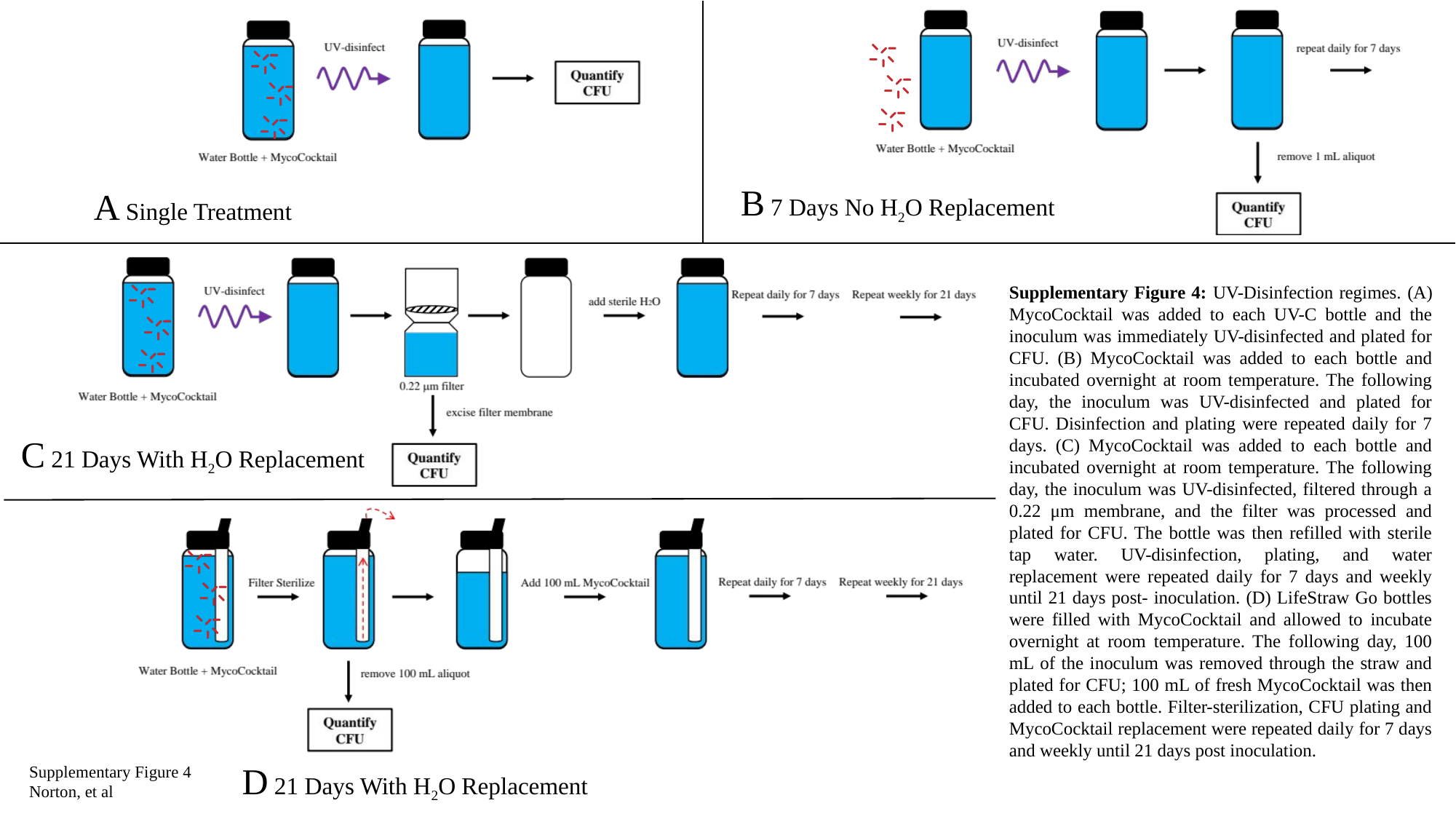

B 7 Days No H2O Replacement
A Single Treatment
Supplementary Figure 4: UV-Disinfection regimes. (A) MycoCocktail was added to each UV-C bottle and the inoculum was immediately UV-disinfected and plated for CFU. (B) MycoCocktail was added to each bottle and incubated overnight at room temperature. The following day, the inoculum was UV-disinfected and plated for CFU. Disinfection and plating were repeated daily for 7 days. (C) MycoCocktail was added to each bottle and incubated overnight at room temperature. The following day, the inoculum was UV-disinfected, filtered through a 0.22 μm membrane, and the filter was processed and plated for CFU. The bottle was then refilled with sterile tap water. UV-disinfection, plating, and water replacement were repeated daily for 7 days and weekly until 21 days post- inoculation. (D) LifeStraw Go bottles were filled with MycoCocktail and allowed to incubate overnight at room temperature. The following day, 100 mL of the inoculum was removed through the straw and plated for CFU; 100 mL of fresh MycoCocktail was then added to each bottle. Filter-sterilization, CFU plating and MycoCocktail replacement were repeated daily for 7 days and weekly until 21 days post inoculation.
C 21 Days With H2O Replacement
D 21 Days With H2O Replacement
Supplementary Figure 4
Norton, et al
